# Differences in Automated External Defibrillator Types in Out-of-Hospital Cardiac Arrest Treated by Police First Responders

**DOI:** 10.3390/jcdd10050196

**Published:** 2023-04-27

**Authors:** Mario Krammel, Jakob Eichelter, Constantin Gatterer, Elisabeth Lobmeyr, Marco Neymayer, Daniel Grassmann, Michael Holzer, Patrick Sulzgruber, Sebastian Schnaubelt

**Affiliations:** 1 PULS—Austrian Cardiac Arrest Awareness Association, 1090 Vienna, Austria; 2 Emergency Medical Service (MA70), 1030 Vienna, Austria; 3 Department of Surgery, Medical University of Vienna, 1090 Vienna, Austria; 4 Division of Cardiology, Department of Internal Medicine II, Medical University of Vienna, 1090 Vienna, Austria; 5 Department of Internal Medicine I, Medical University of Vienna, 1090 Vienna, Austria; 6 Department of Emergency Medicine, Medical University of Vienna, 1090 Vienna, Austria

**Keywords:** automated external defibrillator, out-of-hospital cardiac arrest, basic life support, police, first responder

## Abstract

**Background:** Police first responder systems also including automated external defibrillation (AED) has in the past shown considerable impact on favourable outcomes after out-of-hospital cardiac arrest (OHCA). While short hands-off times in chest compressions are known to be beneficial, various AED models use different algorithms, inducing longer or shorter durations of crucial timeframes along basic life support (BLS). Yet, data on details of these differences, and also of their potential impact on clinical outcomes are scarce. **Methods:** For this retrospective observational study, patients with OHCA of presumed cardiac origin and initially shockable rhythm treated by police first responders in Vienna, Austria, between 01/2013 and 12/2021 were included. Data from the Viennese Cardiac Arrest Registry and AED files were extracted, and exact timeframes were analyzed. **Results:** There were no significant differences in the 350 eligible cases in demographics, return of spontaneous circulation, 30-day survival, or favourable neurological outcome between the used AED types. However, the Philips HS1 and -FrX AEDs showed immediate rhythm analysis after electrode placement (0 [0–1] s) and almost no shock loading time (0 [0–1] s), as opposed to the LP CR Plus (3 [0–4] and 6 [6–6] s, respectively) and LP 1000 (3 [2–10] and 6 [5–7] s, respectively). On the other hand, the HS1 and -FrX had longer analysis times of 12 [12–16] and 12 [11–18] s than the LP CR Plus (5 [5–6] s) and LP 1000 (6 [5–8] s). The duration from when the AED was turned on until the first defibrillation were 45 [28–61] s (Philips FrX), 59 [28–81] s (LP 1000), 59 [50–97] s (HS1), and 69 [55–85] s (LP CR Plus). Conclusion: In a retrospective analysis of OHCA-cases treated by police first responders, we could not find significant differences in clinical patient outcomes concerning the respective used AED model. However, various differences in time durations (e.g., electrode placement to rhythm analysis, analysis duration, or AED turned on until first defibrillation) along the BLS algorithm were seen. This opens up the question of AED-adaptations and tailored training methods for professional first responders.

## 1. Introduction

Outcomes after out-of-hospital cardiac arrest (OHCA) can only be improved by strengthening the so-called chain of survival at any link [1]. Over the last few years, a rise in various first responder systems has be seen, ranging from programs for laypersons to law enforcement or fire brigade personnel. A joint goal is providing basic life support (BLS) including chest compressions and the use of an automated external defibrillator (AED) as soon as possible and, through that, bridging no-flow times until the emergency medical service (EMS) arrives on scene [1,2,3,4,5,6]. An early defibrillation is especially important for favourable outcomes in patients with shockable heart rhythms. Ideas such as equipping police patrol cars with AEDs (=“Pol-AED”) in municipal areas were shown to improve survival and neurological function after cardiac arrest (CA) events, most likely via a much quicker response that would be possible without this system [7,8,9,10,11]. Thinking about room for further improvement, naturally, sufficient and regular training plays an important role [12]. However, another aspect is the scarce data situation on the different types of AEDs used in such systems: Differences in AED workflow and algorithms could mean longer or shorter hands-off-times and, therefore, no-flow times, ergo resulting in an impact on outcome [9,13]. We therefore conducted this retrospective analysis to evaluate potential time- (and thus life-) saving distinctions between the AEDs used in the Viennese Pol-AED program.

## 2. Methods

### 2.1. Study Population

For this retrospective observational study, we included OHCA patients that were treated by police first responders in the city of Vienna, Austria, between January 2013 and December 2021. Inclusion criteria consisted of: >18 years, OHCA with presumed cardiac origin, initially shockable heart rhythm, and police being first on scene starting BLS and delivering the first AED shock. Exclusion criteria were traumatic CA or presumed or actual pregnancy. The timeframe was set from 2013 onwards because the Viennese police first responder program started that year.

This study was conducted as part of the Vienna Cardiac Arrest Registry (VICAR).

Ethical approval for this study was provided by the Ethical Committee of the Medical University of Vienna, Austria (No. 1396/2020). Informed consent was waived. The study protocol complies with the Declaration of Helsinki and data reporting was performed according to the STROBE guidelines.

### 2.2. The Viennese Police First Responder Program

The Pol-AED program in Vienna has already been thoroughly described elsewhere [9]. In brief, the Austrian Cardiac Arrest Awareness Association (PULS) organised AEDs for municipal police cars in Vienna and provided repetitive BLS training for officers in collaboration with the Medical University of Vienna. The AEDs are an addition to standard first-aid equipment like bandages. Such a training (around 60 min) follows guidelines by the European Resuscitation Council [2,12] but adheres to a chest-compression-only policy for simplification and maintenance of high engagement and motivation among the force. The extent of police officers’ CPR training amounted to at least one training during basic police school and, subsequently, at least one follow-up training per year. Since 2013, the number of AEDs in police patrol cars gradually grew, now reaching over 100 in total. Like in other countries [14], the COVID-19 pandemic forced the program to be reduced or even shut down for a limited time, but it was taken up again fast in 2021, as soon as the situation allowed it. During the study period from 2013 to 2021, the applicable ERC guidelines were, respectively, the ones issued in 2010, 2015 and 2021—however, the cornerstones of BLS (chest compressions, AED use) important for our program saw little to no changes, and our training for police officers was conducted without major adaptations.

When an emergency call is placed with the EMS and OHCA is confirmed or suspected, police cars are dispatched there in addition to the emergency physician-based ambulance service. If the police arrive before the EMS, they start BLS and use their AED. The LP (Lifepak) CR Plus (Physio-Control, Redmond, WA, USA), HeartStart FRx (Philips, Amsterdam, The Netherlands), HeartStart HS1 (Philips, Amsterdam, The Netherlands) and LP 1000 (Physio-Control, Redmond, WA, USA) are used by the police force. The choice of the AEDs did not follow a specific protocol but actually depended on governmental acquisition strategies and donations, without the respective companies having any influence on (future) research plans.

### 2.3. Data Acquisition and Patient Follow-Up

All demographic data and CPR details were obtained by specially trained reviewers of the EMS from the event documentation, event time stamps, and the defibrillator file of each case. Data from the AED files were extracted and analyzed via the CODE-STAT Reviewer software package (Physio Control, Redmond, WA, USA) or Philips Heart Start Event Review (Philips, Amsterdam, The Netherlands), and visual evaluation of each ECG file and impedance analysis. In addition, medical professionals then assessed the clinical outcomes via interviews and digital written documentation of the treating physicians. The data were inserted into a predefined case report form, according to Utstein guidelines [15].

### 2.4. Statistical Analysis

The primary outcome was set as neurological function at 30 days after sustained return of spontaneous circulation (sustROSC) as defined by a cerebral performance category (CPC) 1 or 2 [15]. Secondary outcomes included ROSC rates, 30-day survival and various timeframes concerning the EMS or the AEDs. Continuous data are presented as medians and the respective interquartile ranges (IQR) and were compared among subgroups using the Kruskal–Wallis test. Categorical data are presented as counts and percentages and were compared using χ^2^-square tests were appropriate. Statistical significance was defined by two-tailed *p*-values of <0.05. Data analysis was performed using SPSS 22.0 (IBM, Armonk, NY, USA).

## 3. Results

**Figure 1:**
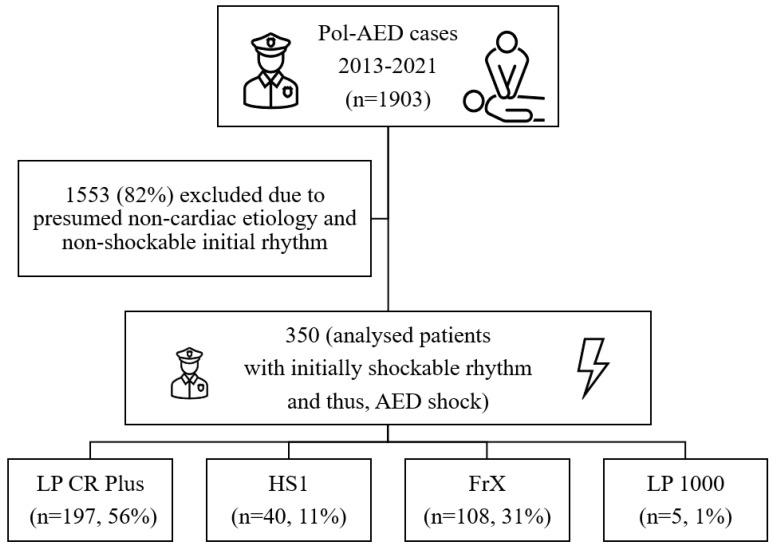
Study flowchart. Pol-AED = police automated external defibrillator. LP (Lifepak) CR Plus: Physio-Control, Redmond, WA, USA; HeartStart FRx: Philips, Amsterdam, Netherlands; HeartStart HS1: Philips, Amsterdam, The Netherlands; LP 1000: Physio-Control, Redmond, WA, USA.

Between January 2013 and December 2021, the police first responders were first on scene in 1903 OHCA events, where they initiated BLS. The AED was used in all cases, at least until rhythm analysis. In 1553 of these episodes, an initially non-shockable rhythm was detected, or the presumed origin of CA was deemed non-cardiac by the emergency physician arriving later on. Thus, 350 cases of OHCA with presumed cardiac etiology, primarily attended by police and showing an initially shockable rhythm when applying the AED, were identified and analyzed (Figure 1).

There were no significant differences in basic demographics, anyROSC, sustROSC, 30-day survival or CPC 1/2 between the cases treated with the different AED types (Table 1).

Timeframes that depict the overall emergency response system in Vienna, such as from the emergency call until EMS arriving on scene, from EMS arriving on scene until the start of advanced life support (ALS), and also from the emergency call until the first delivered shock by a Pol-AED, showed no significant differences when compared in regard to the used AED. Additionally, the times passing between the Pol-AED being turned on and the first chest compression after the first defibrillation, or between the first shock and the first chest compression, did not significantly differ (Table 2).

**Figure 2:**
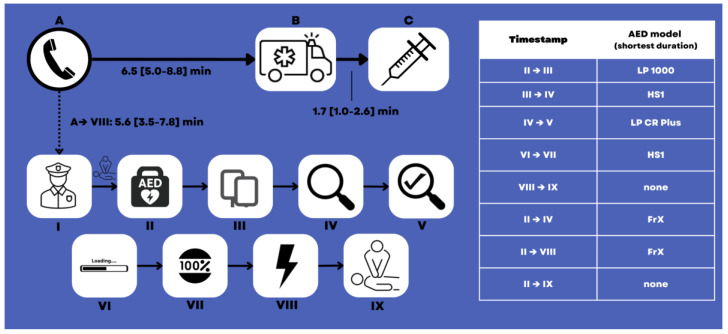
Overview of Pol-AED missions, timeframes during BLS conducted by the police, and the automated external defibrillator (AED) model with the respective shortest durations. (**A**) Emergency call; (**B**) Emergency medical service’ arrival on scene; (**C**) Start of advanced life support; I: Police arriving on scene (no timestamp available); II: AED being turned on (probably with simultaneous chest compressions performed by second police officer until AED is ready); III: Electrodes placed on patient; IV: Start of rhythm analysis; V: Analysis completed; VI: Start of shock loading; VIII: Loading completed; VIII: Shock delivered; IX: Starting chest compressions after the first shock. Additionally, see Table 2. LP (Lifepak) CR Plus: Physio-Control, Redmond, WA, USA; HeartStart FRx: Philips, Amsterdam, The Netherlands; HeartStart HS1: Philips, Amsterdam, The Netherlands; LP 1000: Physio-Control, Redmond, WA, USA.

However, timeframes describing the functioning of the AED itself and/or the immediate handling of the AED did, as a matter of fact, differ between the AED types: The Philips HS1 and -FrX AEDs both showed immediate rhythm analysis after electrode placement (0 [0–1] s) and almost no shock loading time (0 [0–1] s), as opposed to the LP CR Plus (3 [0–4] and 6 [6–6] s, respectively) and the LP 1000 (3 [2–10] and 6 [5–7] s, respectively). On the other hand, the Philips HS1 and -FrX had a longer rhythm analysis time of 12 [12–16] and 12 [11–18] s than the LP CR Plus (5 [5–6] s) and the LP 1000 (6 [5–8] s). The times from the AED being turned on until electrode placement were 12 [4–39] s (LP 1000), 21 [3–37] s (Philips FrX), 35 [29–58] s (Philips HS1), and 43 [31–56] s; turned on to rhythm analysis start 22 [3–38] s (Philips FrX), 39 [10–60] s (LP 1000), 39 [29–80] s (Philips HS1), and 50 [37–66] s (LP CR Plus); and turned on until first defibrillation 45 [28–61] s (Philips FrX), 59 [28–81] s (LP 1000), 59 [50–97] s (Philips HS1), and 69 [55–85] s (LP CR Plus) (Table 2, Figure 2).

## 4. Discussion

After having previously demonstrated the successful implementation of the Viennese Pol-AED program with a considerable effect on patients’ outcomes [9], this study now assessed outcome and event-based timeframe differences between the used AED models. The reported outcome data naturally does not mirror historical lower rates in ROSC, survival, or favourable neurological outcome in Vienna [16], due to the implementation of various first responder systems and other measures in the meantime. Vienna seems to hover midfield when comparing worldwide data [17], always bearing in mind that the analysis at hand represents a highly selected population of presumed cardiac origin of CA, short response times, and an initially shockable rhythm. Of note, this is exactly the primary target population for initiatives such as Pol-AED: Providing help for potentially saveable patients with still good chances of favourable clinical outcomes faster than it would be possible via the EMS alone [9,18,19].

### 4.1. Alternating Stage-Winners during BLS

When compared to historical data, improvements or stable performances concerning the described timeframes can be seen, stressing the applicability of our data [20,21,22].

We could not identify significant differences between the AEDs in general timeframes such as from the emergency call until the first shock, but it was indeed noticeable that once BLS was started and the AED was turned on by the police officers, the models manufactured by Philips and Physio Control outperformed each other in different domains, taking turns in being the fastest (Figure 2).

While difficulties in using AEDs by inexperienced laypersons seem understandable [22,23,24], in the hands of a trained first responder such as the Viennese police force, the use of an AED must be accompanied by prompt and continuous chest compressions, only paused when the AED verbally tells the officers so (rhythm analysis, shock delivery) [2]. These “AED side effects” of algorithm-induced pauses are well-known [21,25]. As any pauses in chest compressions such as peri-shock interruptions mean an interruption in organ perfusion and, thus, enlarging global ischemia [2,26,27], one could identify three main areas of interest concerning “AED speed”: (1) rhythm analysis time, (2) time from shock delivery until resumption of chest compressions, and (3) overall time from the AED being turned on until the first shock (including all other described timeframes). The LP CR Plus “won” in area 1, none of the analyzed devices in area 2 and the FrX in area 3. However, also all other timeframes described in Table 1 and Table 2 and Figure 2 are also of interest due to the human factor behind it: those times will be (at least partly) dependable on the AED-to-user interaction (e.g., listening to voice commands, either waiting for the AED to finish giving orders or working ahead of them, etc.), and the speed of tasks performed by the user (e.g., electrode placing, time to make sure no one is touching the patient before the shock, etc.).

### 4.2. Balanced-Out Effects on Survival and Neurological Function

At the end of the day, clinical patient outcomes are what really counts. We suggest that we could not see significant differences in patient outcomes between the AED models because the “taking turns” in being the fastest in the main areas of interest (see above) could have potentially served as a balancing-out factor between them. The Philips FrX won the “race” to first shock; however, other AEDs probably produced slightly shorter no-flow times.

### 4.3. Future Prospects—Can’t Beggars Be Choosers?

It is known that Pol-AED programs improve patient outcomes after OHCA [7,9,10], and implementation endeavours often include sponsorships or donations in order to be able to acquire a sufficient number of AEDs. However, previous reports and the analysis at hand suggest differences in crucial device features in both artificial and real-life settings that can probably not only be explained through inter-user variability. Even though we could not demonstrate a clear number one among the analyzed models, future research and development work into the respective weak points definitely seem warranted.

In the meantime, one can argue that with intensified first responder AED training, using those AED models that perform better in the machine-induced durations or chest compression pauses [26,28,29].

Typical previously described AED features with room for improvement include large signage, easy opening of the case, better instructions for electrode placement, or more specific general instructions [22,30]. However, those may not apply to regularly trained police first responders—future research is needed here.

Of course, any AED is always better than no AED at all, but maybe beggars should be choosers in terms of demanding improvements from manufacturers, thus inducing increased competition. While outcomes definitely depend on general response times [13], the technical aspect of what comes after arrival on scene must not be shifted out of focus. Technical innovations such as possible chest compressions during rhythm analysis [31] and pre-charging shocks [32] point in the right direction.

## 5. Limitations

This study was performed retrospectively and in a single, high-resource region. Thus, generalization of our findings to other settings and systems is limited. Additionally, the sample size in certain subgroups of AED models was quite low, potentially lacking power to demonstrate differences. As our pool of AED devices was heterogenous, because resources concerning the trainings for police officers were limited, and because police cars carrying a particular AED change with every shift, we could not always train police officers with the specific device model they were going to use in their next real-life scenario. Rather, the training device and the device used in the next CPR were, therefore, allocated randomly. Additionally, we could not provide routine field simulations together with the EMS. Moreover, we did not have data on further patient characteristics, cardiac arrest circumstances, post-resuscitation care, dynamics in outcomes (potentially depicting a learning curve) or more detailed outcomes (e.g., CPC at 90 or 180 days) that could have shown the study circumstances in a more detailed light.

## 6. Conclusions

In a retrospective analysis of 350 cases of out-of-hospital cardiac arrest cases treated by police first responders, we could not find significant differences in clinical patient outcomes concerning the respective used AED model. However, various differences in time durations (e.g., electrode placement to rhythm analysis, analysis duration or AED turned on until first defibrillation) along the BLS algorithm were seen, potentially opening up the question of AED-adaptations and training methods tailored for professional first responders.

## Figures and Tables

**Table 1:** Study population characteristics and clinical outcomes after out-of-hospital cardiac arrest. Categorical data are presented as counts and percentages, continuous data as medians and interquartile ranges (IQR). Kruskal–Wallis tests and chi-square tests were used to assess differences between subgroups. LP (Lifepak) CR Plus: Physio-Control, Redmond, WA, USA; HeartStart FRx: Philips, Amsterdam, The Netherlands; HeartStart HS1: Philips, Amsterdam, The Netherlands; LP 1000: Physio-Control, Redmond, WA, USA; ROSC = return of spontaneous circulation; CPC = cerebral performance category (favourable neurological outcome defined as CPC 1 or 2).

Table 1	Overall (n = 350)	LP CR Plus (n = 197)	Philips HS1 (n = 40)	Philips FrX (n = 108)	LP 1000 (n = 5)	*p*-Value
**Age**, years (IQR)	66 (55–77)	66 (55–77)	67 (56–78)	64 (55–74)	77 (60–83)	0.982
**Male gender**, n (%)	289 (83)	164 (83)	32 (80)	88 (82)	5 (100)	0.812
**anyROSC**, n (%)	200 (57)	116 (59)	20 (50)	63 (58)	1 (20)	0.327
**sustROSC**, n (%)	173 (49)	101 (51)	18 (45)	53 (49)	1 (20)	0.515
**30 days survival**, n (%)	108 (31)	67 (34)	9 (23)	32 (30)	0	0.134
**CPC 1 or 2**, n (%)	91 (26)	56 (28)	7 (18)	28 (26)	0	0.165

**Table 2:** Timeframe durations between crucial Pol-AED resuscitation cornerstones. Categorical data are presented as counts and percentages, continuous data as medians and interquartile ranges (IQR). Kruskal–Wallis tests and chi-square tests were used to assess differences between subgroups. LP (Lifepak) CR Plus: Physio-Control, Redmond, WA, USA; HeartStart FRx: Philips, Amsterdam, The Netherlands; HeartStart HS1: Philips, Amsterdam, The Netherlands; LP 1000: Physio-Control, Redmond, WA, USA; min = minutes; sec = seconds; EMS = emergency medical service; Pol = police; AED = automated external defibrillator.

Table 2	Overall (n = 350)	LP CR Plus (n = 197)	Philips HS1 (n = 40)	Philips FrX (n = 108)	LP 1000 (n = 5)	*p*-Value
**Call** → **EMS on scene**, min (IQR)	6.5 (5.0–8.8)	6.7 (5.2–8.8)	6.0 (4.9–8.7)	6.2 (5.0–8.4)	10.2 (8.6–11.4)	0.991
**EMS on scene** → **ALS start**, min (IQR)	1.7 (1.0–2.6)	1.7 (1.0–2.6)	1.6 (0.8–2.6)	1.8 (1.0–2.6)	1.1 (0.7–1.8)	0.974
**Call** → **Pol-AED turned on**, min (IQR)	4.4 (2.5–6.5)	4.4 (2.6–6.4)	4.7 (2.6–6.8)	4.7 (2.4–6.2)	8.0 (1.0–9.5)	0.995
**Call** → **1st Pol-AED shock**, min (IQR)	5.6 (3.5–7.8)	5.7 (4.0–7.8)	5.6 (3.9–8.4)	5.5 (3.0–7.5)	9.1 (1.7–10.5)	0.201
**Pol-AED turned on** → **electrodes placed on patient**, seconds (IQR)	37 (22–51)	43 (31–56)	35 (29–58)	21 (3–37)	12 (4–39)	**0.003**
**Pol-AED turned on** → **rhythm analysis start**, seconds (IQR)	41 (26–59)	50 (37–66)	39 (29–80)	22 (3–38)	39 (10–60)	**0.022**
**Pol-AED turned on** → **1st shock, seconds**, (IQR)	61 (46–79)	69 (55–85)	59 (50–97)	45 (28–61)	59 (28–81)	**<0.001**
**Pol-AED turned on** → **chest compression start after 1st AED shock**, seconds (IQR)	69 (53–89)	74 (61–94)	70 (61–114)	55 (36–71)	63 (33–88)	0.999
**Electrodes placed on patient** → **analysis start**, seconds (IQR)	3 (0–4)	3 (3–4)	0 (0–1)	0 (0–1)	3 (2–10)	**<0.001**
**Rhythm analysis time**, seconds (IQR)	8 (5–12)	5 (5–6)	12 (12–16)	12 (11–18)	6 (5–8)	**<0.001**
**Shock loading time**, seconds (IQR)	6 (0–6)	6 (6–6)	0 (0–1)	0 (0–1)	6 (5–7)	**<0.001**
**1st POL-AED shock** → **chest compression start**, seconds (IQR)	5 (3–8)	4 (3–6)	7 (3–14)	6 (3–11)	6 (2–7)	0.973

## Data Availability

Data are available from the corresponding author upon reasonable request and consideration of the study team following national legislation.
